# Targeting the Unwindosome by Mebendazole Is a Vulnerability of Chemoresistant Hepatoblastoma

**DOI:** 10.3390/cancers14174196

**Published:** 2022-08-30

**Authors:** Qian Li, Salih Demir, Álvaro Del Río-Álvarez, Rebecca Maxwell, Alexandra Wagner, Juan Carrillo-Reixach, Carolina Armengol, Christian Vokuhl, Beate Häberle, Dietrich von Schweinitz, Irene Schmid, Stefano Cairo, Roland Kappler

**Affiliations:** 1 Department of Pediatric Surgery, Dr. von Hauner Children’s Hospital, University Hospital, LMU Munich, 80337 Munich, Germany; 2 Childhood Liver Oncology Group, Health Sciences Research Institute Germans Trias i Pujol IGTP, 08916 Badalona, Spain; 3 Liver and Digestive Diseases Networking Biomedical Research Centre (CIBEREHD), 28029 Madrid, Spain; 4 Institute of Pathology, University Hospital Bonn, 53127 Bonn, Germany; 5 Department of Pediatrics, Dr. von Hauner Children’s Hospital, University Hospital, LMU Munich, 80337 Munich, Germany; 6 XenTech, 91000 Evry, France

**Keywords:** hepatoblastoma, chemoresistance, connectivity map, mebendazole, unwindosome, gene expression

## Abstract

**Simple Summary:**

Hepatoblastoma patients with tumors that do not respond to preoperative chemotherapy often experience incomplete surgical resection and thus poor outcomes. By analyzing the gene expression data of chemoresistant and responsive tumors using sophisticated drug prediction software we identified mebendazole, a medication usually used to treat parasitic worm infestations, as a putative drug to circumvent chemoresistance. We evaluated the efficacy of this drug in cell culture models of hepatoblastoma and found that both short- and long-term tumor cell growth were significantly inhibited upon treatment. Moreover, we identified the cellular and molecular consequences of mebendazole treatment to be the arrest of cell division and the induction of programmed cell death by the deregulation of genes involved in the unwindosome. Most importantly, mebendazole also proved effective when tested in a mouse model carrying a patient-derived tumor. Our results demonstrate the successful repurposing of mebendazole as a new treatment option for chemoresistant hepatoblastoma.

**Abstract:**

Resistance to conventional chemotherapy remains a huge challenge in the clinical management of hepatoblastoma, the most common liver tumor in childhood. By integrating the gene expression data of hepatoblastoma patients into the perturbation prediction tool Connectivity Map, we identified the clinical widely used anthelmintic mebendazole as a drug to circumvent chemoresistance in permanent and patient-derived xenograft cell lines that are resistant to cisplatin, the therapeutic backbone of hepatoblastoma treatment. Viability assays clearly indicated a potent reduction of tumor cell growth upon mebendazole treatment in a dose-dependent manner. The combination of mebendazole and cisplatin revealed a strong synergistic effect, which was comparable to the one seen with cisplatin and doxorubicin, the current treatment for high-risk hepatoblastoma patients. Moreover, mebendazole treatment resulted in reduced colony and tumor spheroid formation capabilities, cell cycle arrest, and induction of apoptosis of hepatoblastoma cells. Mechanistically, mebendazole causes blockage of microtubule formation and transcriptional downregulation of genes encoding the unwindosome, which are highly expressed in chemoresistant tumors. Most importantly, mebendazole significantly reduced tumor growth in a subcutaneous xenograft transplantation mouse model without side effects. In conclusion, our results strongly support the clinical use of mebendazole in the treatment of chemoresistant hepatoblastoma and highlight the potential theranostic value of unwindosome-associated genes.

## 1. Introduction

Hepatoblastoma (HB) is the most common primary hepatic malignancy in children, occurring almost exclusively under the age of 5 years [1]. Patients commonly present with an abdominal mass found on ultrasound and pathologically elevated levels of serum alpha-fetoprotein (AFP). In contrast to hepatocellular carcinoma, which develops in older patients in the setting of cirrhosis and hepatitis, HB generally occurs without any underlying liver pathologies. Although HB is associated with some genetic predisposition syndromes, such as familial adenomatous polyposis, Beckwith–Wiedemann, Simpson–Golabi Behmel, and Sotos syndromes, nearly all cases occur sporadically [2]. Genome-wide studies have shown an overall very low mutation rate with only a few recurrent mutations such as the one in ß-catenin, thus leaving the genetic basis of sporadic HB mainly elusive [3,4,5,6]. The survival rate of HB patients has dramatically increased over the last 30 years, largely due to improvements in chemotherapy, surgical techniques, and the introduction of orthotopic liver transplantation for locally unresectable disease. Contemporary studies have revealed a three-year overall survival rate of 83% in standard-risk patients treated with a combination of cisplatin monotherapy and surgery [7]. However, for patients with high-risk features, such as metastatic disease or a tumor involving all four liver sections, or for whom even after alternating cycles of cisplatin and doxorubicin a radical surgical resection is not possible, the prognosis still remains poor [8]. Thus, resistance to chemotherapy remains a huge challenge in the clinical management and treatment of HB, and novel agents and therapeutic options for these patients are urgently needed.

Repurposing of drugs with known pharmacokinetics and safety profiles that have previously been approved for other indications is a promising strategy to reduce the cost and time required to develop new anti-cancer drugs. The Connectivity Map (CMap) database has been developed to link gene expression patterns associated with a distinct phenotype or disease to corresponding patterns derived from drug-treated cancer cell lines [9,10]. CMap has already been successfully used to identify potential new treatment options in a variety of cancers [11,12,13]. For HB, there is extensive transcriptomic data that has been used to describe specific expression patterns associated with the biology and clinical characteristics of this tumor [4,5,6,14,15,16]. These studies clearly revealed three main prognostic subclasses of tumors displaying strong differences in their gene expression profile in terms of stem-cell/progenitor-like/proliferation markers, hepatic function, and mesenchymal characteristics. The prognostic relevance of the so-called 16-gene signature [14] has recently been validated in a large cohort of HB patients, thus leading to the proposal that biological and clinical factors should be combined so as to improve the risk-adapted management of HB patients [17]. However, none of these studies provided data on the treatment modalities and response to chemotherapy of the patients.

In our study, we integrated RNA sequencing-derived expression data of HB patients [18] treated according to the German HB99 protocol [19] into the CMap tool [9] and identified the clinical widely used anthelmintic mebendazole as a putative drug to circumvent chemoresistance in HB. We then showed that mebendazole is able to reduce tumor growth in HB cells by arresting the cell cycle in the G2/M phase and inducing apoptosis. Moreover, we also revealed a novel therapeutic synergy between cisplatin and mebendazole, which was comparable to the one seen with cisplatin and doxorubicin. Most importantly, we demonstrated that in vivo growth of a patient-derived xenograft tumor was significantly impeded by oral mebendazole application in mice. These findings suggest that mebendazole is a potential new therapy option for chemoresistant HB.

## 2. Materials and Methods

### 2.1. Connectivity Map Analysis

Data were downloaded from Gene Expression Omnibus with the accession number GSE151347. Read counts obtained from RNA sequencing data were normalized and analyzed for differentially expressed genes between 2 non-responders and 5 responders using the Bioconductor package DESeq2 [20]. A decline in AFP level ≥ 1 log10 after two cycles of chemotherapy was considered indicative of tumor responsiveness to chemotherapy, as previously described [19]. The most significantly deregulated genes (*p*-value < 0.01) (Supplementary Table S1) from the chemoresistant HB patients as compared to the responders were identified. These genes were submitted as a query signature to the extended CMap Build 02 tool “http://www.broadinstitute.org/cmap (accessed on 15 February 2019)”. This tool then assessed the similarity of this signature to each of the reference expression profiles in the database. These profiles have been generated by treating the four human cancer cell lines MCF7, PC3, HL60, and SKMEL5 with 1309 therapeutic compounds at varying concentrations and time points [21]. Positive connectivity scores indicate that drugs are able to induce the input signature in human cell lines. Negative connectivity scores specify that drugs could reverse the input signature, thus indicating a potential therapeutic value.

### 2.2. Cell Lines

Established cell lines were composed of the three HB cell lines HUH6 (Japanese Collection of Research Bioresources, Osaka, Japan), HepG2 (ATCC, Manassas, VA, USA), and HepT1 [22], as well as the two hepatocellular carcinoma cell lines Hep3B (ATCC) and HUH7 (Japanese Collection of Research Bioresources). All cell lines were incubated in RPMI 1640 (Life Technologies, Carlsbad, CA, USA) supplemented with 10% (*v/v*) fetal bovine serum (Life Technologies) and 1% (*v/v*) penicillin/streptomycin (Invitrogen, Waltham, MA, USA). We also used seven HB cell lines generated from patient-derived xenograft (PDX) models (XenTech, Evry, France) [23]. All PDX cell lines were maintained in advanced DMEM/F12 (Life Technologies) supplemented with 10% (*v/v*) fetal bovine serum, 1% (*v/v*) penicillin/streptomycin (Invitrogen), 1% (*v/v*) L-glutamine (Thermo Fisher, Waltham, MA, USA), and the Rho-associated kinase inhibitor Y-27632 (Selleckchem, Chesterbrook, PA, USA) at a final concentration of 20 µM.

### 2.3. Viability Assay

MTT (3-(4,5-dimethylthiazol-2-yl)-2,5-diphenyltetrazolium bromide) (Sigma-Aldrich, St. Louis MO, USA) viability assays were performed for determining drug responses of the cells. 1 × 10^4^ cells/well were seeded in 96-well plates 24 h prior to drug exposure. All cell lines and PDXs were exposed to DMSO as a control or seven increasing concentrations of mebendazole (Selleckchem) ranging from 0.128 nM to 20 µM as 1:5 serial dilutions. Absorbance values were measured with the Sunrise plate reader (Tecan, Männedorf, Switzerland) after 48 h incubation, and half-maximal inhibitory concentrations (IC_50_) were calculated using GraphPad Prism 8 software (GraphPad Software, San Diego, CA, USA).

### 2.4. Synergy Assay

Pairwise drug combinations were tested for synergistic effects using MTT-based cell viability data of tumor cell lines exposed for 48 h to four different concentrations of either cisplatin and doxorubicin or cisplatin and mebendazole (all from Selleckchem). Maximum synergy scores and the corresponding concentrations were determined using the HSA statistical reference model of the Synergyfinder 2.0 software [24].

### 2.5. Proliferation Assay

The click-iT EdU cell proliferation kit (Thermo Fisher) was applied according to manufacturer’s instructions for the detection of proliferating cell portions. In brief, 1 × 10^5^ cells/well were seeded in 24-well plates and cultured overnight before treatment. After refreshing media on the next day, cells were labelled with 100 µM ethynyl deoxyuridine (EdU) and subsequently exposed to mebendazole (Selleckchem) or dimethyl sulfoxide (DMSO) for 24 h at 37 °C. After 3.7% paraformaldehyde/PBS fixation and 0.5% Triton-X permeabilization, cells were stained with Alexa Fluor 555 azide for 30 min. Hoechst 33342 was used for staining of nuclei. Images were captured with the 10× objective of the EVOS M7000 microscope (Invitrogen) and EdU-positive nuclei counted in relation to the total number of Hoechst 33342-positive nuclei using the EVOS software.

### 2.6. Colony Formation Assay

1 × 10^4^ cells/well were seeded in 6-well plates and incubated overnight. The next day, cells were changed into culturing media containing either DMSO or 0.5 µM mebendazole for 5 days. Upon formation of colonies, cells were fixed with ice-cold methanol for 15 min and stained with 0.5% crystal violet (Sigma-Aldrich) in 20% methanol for 1 h. Pictures were taken with using the 4× objective of the EVOS M7000 microscope (Invitrogen) and colonies irrespective of their size counted with ImageJ “https://imagej.nih.gov/ij/” (accessed on 25 April 2021).

### 2.7. Spheroid Formation Assay

For the determination of the spheroid forming capability of tumor cell lines, 1 × 10^3^ cells were seeded into ultra-low attachment round bottom 96-well plates (Corning, Corning, NY, USA) in culturing medium containing either DMSO or mebendazole. After 4 days, spheroid images were captured by EVOS M7000 microscope (Invitrogen). In order to investigate the spheroid volume changes upon treatment, 1 × 10^3^ cells were first seeded into ultra-low attachment round-bottom 96-well plates and then cultured for 7 days until spheroids were established. Mebendazole or DMSO exposure was initiated and images were captured at days 0, 4, and 7 with the 10× objective of the EVOS M7000 microscope (Invitrogen). Spheroid volumes (V) were calculated by V (µm^3^) = [(length (µm) × width (µm)^2^)/2] formula [25].

### 2.8. Cell Cycle Analysis

Cell cycle analysis was performed by Hoechst 33342 staining. For synchronization, cells were cultured in starvation medium without serum for 24 h. Then, serum-free medium was replaced with normal media and the cells were exposed to DMSO or mebendazole for 48 h. 150,000 cells were stained with 1 µg/mL Hoechst 33342 (Invitrogen) in modified advanced DMEM/F12 medium in the dark at 37 °C for 30 min. The distribution of cell cycle phases was measured by the LSRFortessa cell analyzer (BD Biosciences, East Rutherford, NJ, USA) and data analysis was done by FlowJo software v10.4 (BD Biosciences).

### 2.9. Immunofluorescence

Briefly, 2 × 10^5^ cells were seeded in µ-slide 8-well chambered coverslips (Ibidi, Gräfelfing, Germany) and incubated in culturing medium containing either DMSO or mebendazole for 24 h. In addition, 5 µm cryosections of tumor samples of the animal studies that have been air-dried for 10 min were used. Cells or tissue sections were fixed in 4% paraformaldehyde/PBS for 15 min, permeabilized with 0.5% Triton X-100 for 1 h and then blocked in PBST containing 3% BSA and 0.1% glycine for 10 min. Slides were then incubated overnight at 4 °C with mouse anti-α-tubulin (clone DM1A) (Sigma-Aldrich), Ki67 (Abcam, Cambridge, UK) and cleaved caspase-3 (Cell Signaling Technology, Danvers, MA, USA) antibodies in 5% BSA/PBS at dilutions of 1:100, 1:250, and 1:100, respectively. The next day, following serial washing steps, slides were incubated with Alexa Fluor 555-conjugated goat anti-rabbit IgG secondary antibody (Invitrogen) in a dilution of 1:250 in 5% BSA and 0.1% Triton X-100. Slides were counterstained with 1 µg/mL Hoechst 33342 (Invitrogen) at room temperature in the dark for 1 h. Images of tubulin staining were captured with the 40× objective of the Axiovert 200M microscope equipped with an AxioCam MRm (Zeiss, Jena, Germany) and abnormal spindles were counted by ImageJ. Ki67 and caspase-3 stainings were captured with the EVOS M7000 microscope (Invitrogen).

### 2.10. Apoptosis Assay

Detection of apoptotic cells by flow cytometry was performed using Annexin V/propidium iodide staining according to the manufacturer’s instructions; 1.5 × 10^4^ cells were rinsed with ice-cold 1× Annexin binding buffer (Invitrogen) and 100 μL of 1:20 APC-conjugated Annexin V (BD Biosciences) solution was added to the cells and incubated for 60 min at room temperature. Following 20 ng/mL propidium iodide incubation for 5 min at room temperature, immediate acquisition was performed on the LSRFortessa cell analyzer (BD Biosciences). Cells undergoing apoptotic cell death were determined using FlowJo software v10.4 (BD Biosciences) by calculating the portions of Annexin V positivity.

### 2.11. RNA-Sequencing

Total RNA was isolated from each of the DMSO or mebendazole-treated PDX cell lines using TRIzol (Invitrogen). Libraries were prepared from 1 µg total RNA with the TruSeq stranded mRNA kit (Illumina, San Diego, CA, USA). Sequencing was done on a HiSeq2500 system (Illumina) as 100 bp paired-end runs generating 35–83 million mapped reads. Split-read alignment against the human genome assembly hg19 (GRCh37) and UCSC known gene annotation was accomplished using STAR aligner v2.4.2a [26] with modified parameter settings (--twopassMode = Basic). Number of reads mapping to annotated genes were quantified using HTseq-count v0.6.0 [27]. Read counts obtained from RNA-sequencing data were normalized and analyzed for differential expressed genes between treated and untreated cell lines by using the Bioconductor package DESeq2 [20]. Data are available at Gene Expression Omnibus (accession number GSE209824). For gene set enrichment analysis, we used the ShinyGO 0.76 package [28] and differently expressed genes > 2-fold with a *p*-value < 0.05 as an input. Results were ranked according to the false discovery rate based on nominal *p*-value from the hypergeometric test and then sorted by fold enrichment [28]. For protein–protein interaction mapping, the STRING 11.5 tool was used [29].

### 2.12. Animal Studies

Animal studies were carried out by XenTech as previously described [23] under the license for experiments on vertebrate animals, issued by the French Ministry of Higher Education, Research and Innovation (APAFIS#29136-2020121415204532, 15 January 2021). Cryopreserved tumor pieces of the PDX model HB-282 were implanted in the interscapular region of 5-week-old female nude-Foxn1nu mice (Hsd:Athymic Nude-Foxn1nu, ENVIGO, Gannat, France). After a latency period, mice with a subcutaneously growing tumor between 75 and 288 mm^3^ were allocated to each treatment arm according to their tumor volume, so as to maintain homogenous mean and median tumor volume in each arm, with seven mice per group. Mice were treated with vehicle or 40 mg/kg mebendazole (suspension in PBS) by oral administration for 5 days on and 2 days off. The length and the width (defined by the longest and the shortest diameters of the tumor, respectively) were measured with a caliper three times a week during the latency and treatment periods. Tumor volume (TV) was calculated using the formula: TV (mm^3^) = [(length (mm) × width (mm)^2^)/2]. All animals were weighed at tumor measurement time and were observed every day for physical appearance, behavior, and clinical changes. The ratio between the mean TV of the mebendazole-treated and the control group was calculated at each measurement.

Three tumor samples per group were resected and snap-frozen. Cryosections of 5 µm thickness were fixed for 10 min in 4% paraformaldehyde/PBS and stained with hematoxylin (Roth, Karlsruhe, Germany) and eosin (Sigma-Aldrich) for 4 min and 15 s, respectively. Slides were subsequently dehydrated through serial steps of 70%, 95%, and 100% ethanol and then mounted in RotiHistol (Roth) before images were captured on the EVOS M7000 microscope (Invitrogen).

### 2.13. Statistical Analyses

Statistical analyses were conducted with GraphPad Prism 8 (GraphPad Software). Data are expressed as mean ± standard error of the mean (SEM) or standard deviation (SD). For all assays, differences between two groups were analyzed by Student’s *t* test, whereas differences between more than two groups were analyzed by ANOVA test with Tukey post-hoc test. For event-free (EFS) and overall (OS) survival analysis, Kaplan–Meier’s method and log-rank tests were performed to compare differences between curves. Tumor samples were scored high if at least 3 of the 4 unwindosome genes had higher expression levels than the median of each gene. Values of *p* < 0.05 were considered significant for all analyses.

## 3. Results

### 3.1. Connectivity Map Identifies Mebendazole as a Candidate Drug for Chemoresistant Hepatoblastoma

**Figure 1:**
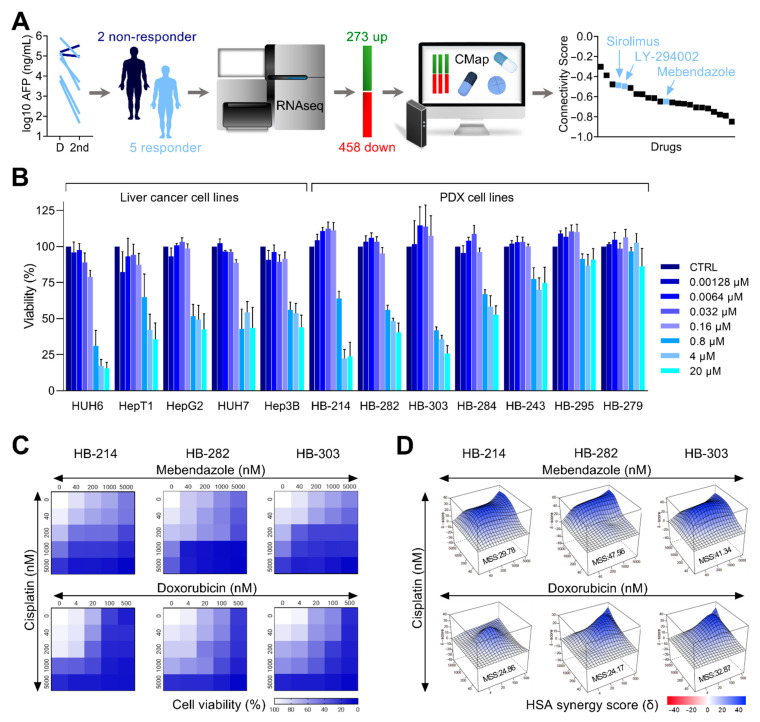
Repurposing of mebendazole for chemoresistant hepatoblastoma. (**A**) Alpha-fetoprotein (AFP) levels in the serum of patients were measured at diagnosis (D) and after the second cycle of chemotherapy (2nd) and are given as log10 ng/mL. Responsiveness to chemotherapy was considered as a decline in AFP level log10 ≥ 1. RNA sequencing data from the 2 non-responders and the 5 responders were retrieved from the Gene Expression Omnibus and differentially expressed genes integrated into Connectivity Map (CMap). The graph shows candidate drugs predicted by CMap with a negative connectivity score indicative of a potential therapeutic value. (**B**) MTT-based viability assays displaying the response of 5 liver cancer cell lines and 7 PDX cell lines towards mebendazole exposure with 7 increasing concentrations ranging from 0.00128–20 µM. Error bars represent standard error of the mean (±SEM) of three independent experiments, each consisting of two replicates. (**C**) Heatmaps displaying the proportions of cell viability upon exposure towards increasing concentrations (given in nM) of cisplatin, mebendazole, and doxorubicin in a two-drug combination matrix format (n = 2, duplicates). (**D**) Three-dimensional synergy landscapes shown separately for each two-drug combination of cisplatin, mebendazole, and doxorubicin, as calculated from cell viability assays (n = 2, duplicates). Maximum synergy scores (MSS) were calculated via Synergyfinder2.0 software with the HSA model.

In order to identify drugs that could be used to circumvent chemoresistance in HB, we made use of the CMap drug prediction tool [21] and a RNAseq-derived gene expression dataset on a small cohort of HB patients [18]. This cohort of patients had been treated according to the German HB99 protocol [19] and there was therapy response data available. Five of the seven patients had responded to preoperative chemotherapy with a decrease in AFP ≥ 1 log10 during the first two cycles of chemotherapy [19]. By comparing global gene expression levels of the two non-responders and the five responders, we identified 273 upregulated and 458 downregulated genes (Supplementary Table S1) as being differentially expressed in the non-responders (Figure 1A). These differentially expressed genes then constituted a gene signature, which was used as an input query for CMap to identify compounds with negative correlation scores, implying potential to evade chemoresistance in HB. From the 1309 perturbagens contained in CMap, we identified 23 candidate drugs (Supplementary Table S2, Figure 1A), of which two drugs have already been shown previously to impede growth of HB cells, namely sirolimus [30] and LY-294002 [31], thereby underscoring the predictive power of this approach. In addition, we found the clinical widely used anthelmintic drug mebendazole as another candidate, showing an even stronger negative correlation score as the aforementioned medications.

Our next step was to experimentally validate the efficacy of the newly predicted drug mebendazole in different cell culture models of liver tumors [23]. We were able to show that mebendazole did indeed decrease cell viability in a dose-dependent manner in five established and widely used permanent liver tumor cell lines [22,32,33] in short-term cultures (Figure 1B). Moreover, using cell lines generated from PDX tumors of an independent cohort of HB patients [34], which more closely display patient-near modeling of the disease, we found that five out of seven cell lines responded to mebendazole treatment (Figure 1B).

As cisplatin is the backbone of the standard therapy for HB [35], we then wanted to see how a combination of mebendazole with cisplatin acts on the viability of liver tumor cells. To do so, we focused on the three PDX cell lines HB-214, HB-282, and HB-303, which showed the highest sensitivity to mebendazole, with clinically achievable IC50 values of 1.63 µM, 4.53 µM, and 1.59 µM, respectively. The combination of both drugs resulted in a striking decrease of cell viability even at the nanomolar level (Figure 1C), which was comparable to the one seen with cisplatin and doxorubicin, the standard of care for high-risk HB patients [35]. Moreover, by calculating the maximal synergy score for varying concentrations of the drugs, we found comparable or even higher synergies of mebendazole and cisplatin, as compared to the cisplatin and doxorubicin combination (Figure 1D), thereby suggesting that mebendazole might be a better choice to combine with cisplatin. Altogether, our data clearly show that CMap is a powerful tool for the repurposing of drugs for chemoresistant HB.

### 3.2. Mebendazole Inhibits the Proliferation of Hepatoblastoma Cells by Spindle Disruption and Cell Cycle Arrest

**Figure 2:**
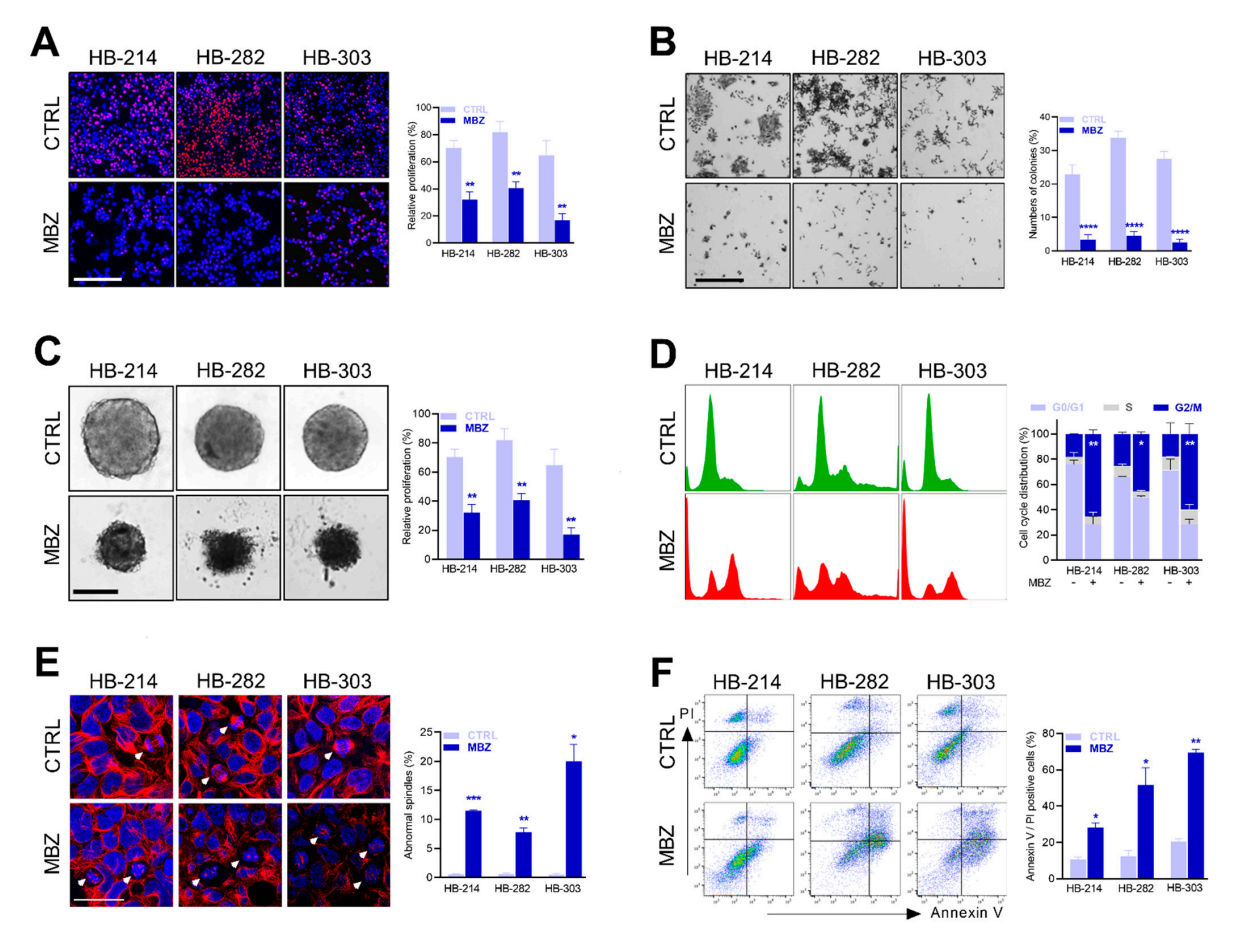
Mebendazole hinders short and long-term cell growth due to cell cycle arrest. (**A**) Proliferating cells were detected by EdU-staining (red) and quantified in relation to Hoechst 33342-stained nuclei (blue). HB-214, HB-282, and HB-303 cells were exposed to DMSO (CTRL) or mebendazole (MBZ) in a concentration of 2 μM, 4 μM, and 2 μM, respectively. The graph represents the mean of 3 experiments. Scale bar represents 300 μm. (**B**) Long-term growth of the PDX cells was detected by colony formation assay. Cells were exposed to DMSO or 0.5 μM mebendazole for 7 days, and colonies were stained with crystal violet. The graph represents the mean of 3 experiments. Scale bar represents 750 μm. (**C**) The capacity of PDX cells forming three-dimensional tumor spheroids was monitored on day 4 after exposure to 2 μM, 4 μM, and 2 μM mebendazole. The graph represents the mean spheroid volumes of 3 experiments. Scale bar represents 300 μm. (**D**) Distributions of cell cycle phases were determined on Hoechst 33342-stained cells treated with 2 μM, 1 μM, and 2 μM mebendazole for 48 h by flow cytometry acquisition. The graph represents the mean of 2 independent experiments analyzed by FlowJo. (**E**) Mitotic spindles were detected by alpha-tubulin (TUBA) staining (red) in Hoechst 33342-counterstained nuclei (blue) of mebendazole exposed cells (24 h; 2 μM, 4 μM, and 2 μM). The arrows indicate normal spindles in the CTRL group and abnormal spindles in the MBZ group. The graph displays the mean of 2 independent experiments. Scale bar represents 50 µm. (**F**) Apoptosis was detected by Annexin V/propidium iodide double-stain via flow cytometry in MBZ exposed cells (48 h; 2 μM, 4 μM, and 2 μM). The graph represents the mean of 2 independent experiments. For all experiments in (**A**–**F**), error bars represent standard deviation (±SD), *p*-values were calculated using a two-tailed unpaired Student’s *t* test and significance determined as follows: ns, not significant; * *p* < 0.05; ** *p* < 0.01; *** *p* < 0.001, **** *p*< 0.0001.

Next, we tried to elucidate the cellular consequences of mebendazole treatment in liver tumor cells. The fluorescence microscopic analysis of the cell lines treated with mebendazole revealed a dramatic decrease of proliferating cells compared to untreated controls (Figure 2A). Moreover, colony formation assays clearly indicated that the reproductive integrity of tumor cells was adversely affected in long-term cultures, as the cell lines formed only a few colonies upon treatment (Figure 2B). In addition, the potential of all three cell lines to form three-dimensional tumor spheroids was heavily impacted when grown in mebendazole-containing media, as evidenced by irregular shaped, poorly organized, and small cell aggregates (Figure 2C). A subsequent flow cytometry-based cell cycle analysis revealed a strong mebendazole-induced arrest of tumor cells in the G2/M phase (Figure 2D), thereby suggesting that a halt of tumor cells during cell division is a plausible mode of action by which mebendazole causes growth inhibition.

As it is known that mebendazole can interfere with the assembly of tubulins that are vital to cell division [36], we fluorescently stained mebendazole-treated liver cancer cells for α-tubulin, a major component of the mitotic spindle. We found that mebendazole dramatically induced disrupted mitotic spindles in the three selected PDX cell lines (Figure 2E). Using flow cytometric apoptosis assays, we furthermore were able to show that mebendazole treatment triggered the programmed cell death in these cell lines, as demonstrated by a high proportion of Annexin V and propidium iodide double-positive cells (Figure 2F). Collectively, our results clearly indicate that mebendazole inhibits the short- and long-term proliferation of HB cells by promoting cell cycle arrest, which is caused by defective mitotic spindles, thereby ultimately leading to apoptosis.

### 3.3. Mebendazole Drives Deregulation of Genes Associated with the Cell Cycle and the Unwindosome

**Figure 3:**
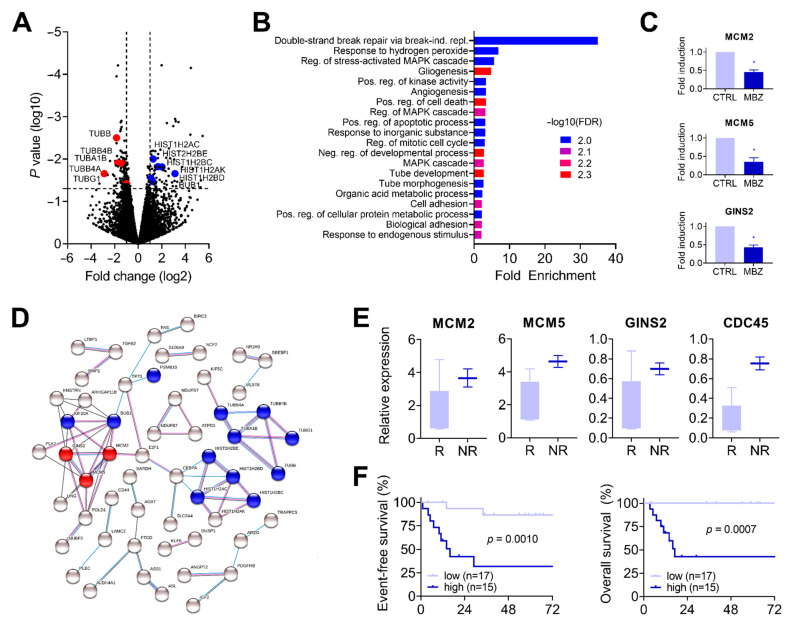
Mebendazole treatment alters expression of unwindosome genes. (**A**) Volcano plot of RNA expression data of HB-214, HB-282, and HB-303 cells exposed for 24 h to DMSO or mebendazole at concentrations of 2 μM, 4 μM, and 2 μM, respectively. Transcriptionally up-regulated candidate genes are depicted in blue, down-regulated genes in red. (**B**) Gene ontology analysis of differentially expressed genes with *p*-value < 0.05 displaying enrichment in cellular pathways. (**C**) Mean fold induction of RNA expression of the three genes contained in the top-scoring term “double-strand break repair via break-induced replication” in the DMSO (CTRL) and mebendazole (MBZ) treated tumors cell lines HB-214, HB-282, and HB-303. Error bars represent standard deviation, with * being indicative for *p* < 0.05 calculated from Student’s *t* test. (**D**) Protein-protein interaction network analysis of mebendazole regulated candidate genes. The nodes and edges represent query genes and relationships between candidates, respectively. Components of the unwindosome are highlighted in red, components of spindle/chromosome-associated cell division in blue. Blue edges indicate gene co-occurrence, black edges gene co-expression, and purple edges experimentally determined interactions. (**E**) Relative RNA expression of unwindosome genes in patient samples that responded (R) and did not respond (NR) to chemotherapy (retrieved from the Gene Expression Omnibus GSE151347 data set). (**F**) Event-free and overall survival Kaplan–Meier plots of high versus low unwindosome-expressing tumors according to the median. Expression data were retrieved from the Gene Expression Omnibus with the accession number GSE132219 [15].

To obtain a more global view of the functional role of mebendazole treatment on HB, we exposed HB-214, HB-282, and HB-303 cells to mebendazole for 24 h and subjected them to RNA sequencing. The comparison of the global expression levels of the three treated with the three untreated PDX lines revealed 74 and 115 genes to be significantly (*p* < 0.05) induced and repressed > 2-fold, respectively (Figure 3A, Supplementary Table S3). In agreement with the disrupted mitotic spindles found before (Figure 2E), we detected transcriptional downregulation of the two components constituting microtubules (Figure 3A), namely alpha-tubulin (*TUBA1B*) and beta-tubulin (*TUBB, TUBB4A, TUBB4B*), as well as gamma-tubulin (*TUBG1*), which organizes the ring complexes for microtubule nucleation [37]. In contrast, we found genes coding for the histone H2A (*HIST1H2AC, HIST1H2AK*) and H2B families (*HIST1H2BC, HIST1H2BD, HIST2H2BE*) to be significantly upregulated (Figure 3A). These histones are located at centromeres and ensure appropriate chromosome separation [38]. In addition, the mitotic checkpoint serine/threonine kinase (*BUB1*), which phosphorylates histone H2A at the centromere and participates in the spindle assembly checkpoint that prevents precocious separation of sister chromatids [39] was found to be transcriptionally upregulated. Altogether, our RNA sequencing data suggest that the regulatory cascade of spindle attachment and chromosome segregation are heavily affected by mebendazole treatment.

In order to identify other biological processes apart from the spindle/chromosome-associated cell division that relate to mebendazole treatment, we applied gene ontology (GO) enrichment and protein–protein interaction network analysis to the differentially expressed genes detected by RNA sequencing (Figure 3B,C). In agreement with the previous in vitro findings, we detected cell cycle (GO:0007346) and apoptosis (GO:0010942 and GO:0043065) related gene enrichment within the top 20 ranking categories (Figure 3B, Supplementary Table S4). GO enrichment analysis revealed the GO term “double-strand break repair via break-induced replication” (GO:0000727) as the highest scoring category by far, containing the genes of the minichromosome maintenance complex components 2 and 5 (*MCM2, MCM5*) and the GINS complex subunit 2 (*GINS2*). Further analysis of the RNA sequencing data from the treatment experiments clearly highlighted that these genes were strongly downregulated in all three PDX lines upon mebendazole treatment (Figure 3C). Interestingly, GINS2 and CDC45 proteins together with other MCM subunits build up the so-called unwindosome, which assembles during cell cycling in order to unwind duplex DNA during DNA replication [40]. The creation of a protein–protein interaction network of candidate genes underscored the functional relationship between unwindosome components and spindle/chromosome-associated cell division (Figure 3D).

In order to assess if unwindosome activation could be indicative of resistance to chemotherapy, we went back to the initial RNA sequencing data and profiled the expression level of the respective genes. Notably, we could indeed corroborate that *MCM2, MCM5, GINS2*, and *CDC45* mRNA levels were highly increased in tumors that did not respond to chemotherapy as compared to responders (Figure 3E). Next, we wanted to investigate whether transcriptional upregulation of these genes is associated with other clinical and molecular characteristics. By evaluating RNA expression data of 32 clinically annotated HB patients of a former study [15] we were able to show that high expression of *MCM2, MCM5, GINS2*, and *CDC45* was not associated with any clinical annotation factor, such as metastasis, vascular invasion, age > 8 years, or PRETEXT 4 stage (Table 1). However, transcriptional activation of unwindosome genes was significantly associated with two molecular risk factors for HB (Table 1), namely the adverse C2 subtype of the 16-gene signature [14] and the high-risk MRS-3 subtype [15], both of which have been reported as being associated with poor outcomes. Accordingly, when patients were stratified according to their unwindosome activation we found a significantly poorer event-free and overall survival in high-expressing unwindosome patients (Figure 3F). These data clearly underscore the importance of unwindosome-associated genes in conveying chemoresistance and poor outcome in HB.

### 3.4. Mebendazole Treatment Inhibits Tumor Growth in Patient-Near Spheroid and Xenograft Models

**Figure 4:**
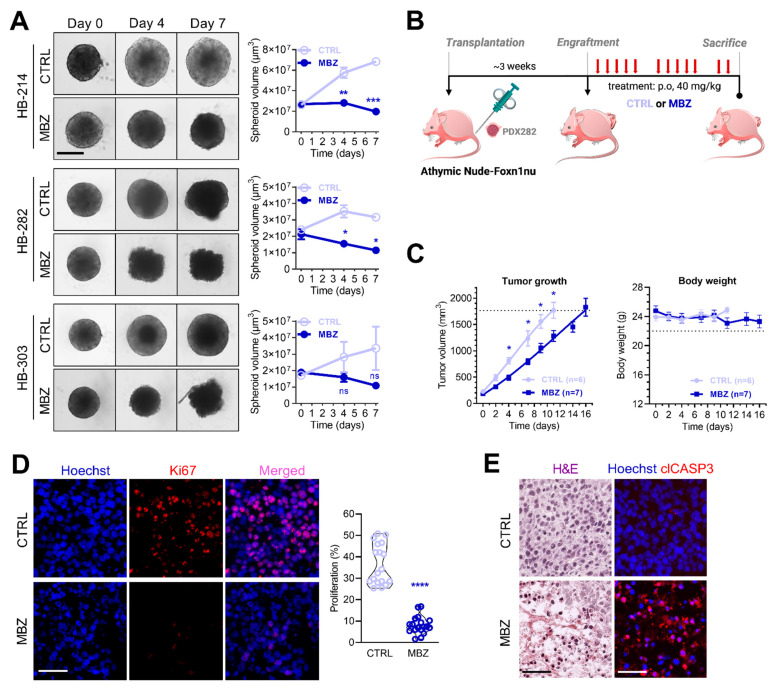
Mebendazole testing in patient-near spheroid and xenograft models. (**A**) Established tumor spheroids of HB-214, HB-282, and HB-303 cells were treated with vehicle (CTRL) or mebendazole (MBZ) at concentrations of 2 μM, 4 μM, and 2 μM for 0, 4, and 7 days. The graph represents mean spheroid volumes of one experiment consisting of triplicate measurements. Scale bars represent 300 μm, error bars represent ± SD. *p*-values were calculated using a two-tailed unpaired Student’s *t* test. (**B**) Experimental overview of mebendazole testing in vivo. Immunocompromised mice bearing subcutaneously transplanted HB-282 tumors were treated with 40 mg/kg body weight mebendazole (MBZ) by oral administration or vehicle (CTRL) 5 times per week. Mice were sacrificed on the day that they reached the maximal tolerable tumor size of 1,764 mm^3^. (**C**) Tumor growth (left panel) and body weight changes (right panel) in mice treated with either MBZ (n = 7) or vehicle (n = 6). Values correspond to mean tumor volumes and mean body weights ± SEM. Dashed lines indicate maximum permissible values. (**D**) Immunofluorescent staining of Ki67 positive nuclei (red) counterstained with Hoechst 33342 (blue) of vehicle (CTRL) and mebendazole (MBZ) treated tumors. Scale bars represent 50 µm. Proliferation was calculated by relating Ki67 positive nuclei of six representative areas of three tumors per condition to Hoechst 33342 nuclei. (**E**) Hematoxylin/eosin (H&E) staining of vehicle (CTRL) and mebendazole (MBZ)-treated tumors (**left** panel) and immunofluorescent staining of cleaved caspase-3 (clCASP3; red) indicating apoptotic regions counterstained with Hoechst 33342 (**right** panel). Scale bars represent 50 µm. Significance is given as follows: ns, not significant; * *p* < 0.05; ** *p* < 0.01; *** *p* < 0.001; **** *p* < 0.0001.

As three-dimensional cultures mimic the native tumor microenvironment, such as cell–cell and cell–extracellular matrix interactions [43], we tested whether mebendazole could also impact growth of tumor cells after they have already formed large tumor spheroids. We found that in the absence of mebendazole, tumor spheroids were still able to grow further, whereas treatment resulted in not only inhibition, but also a reduction of the spheroid volume (Figure 4A).

To directly investigate the efficacy of mebendazole in the preclinical setting, we used an established patient-derived xenograft model in which HB-282 tumors were transplanted into immunocompromised mice. We selected this PDX line because this model had the greatest sensitivity to mebendazole in our in vitro studies (Figure 1B) and displayed an aggressive growth in vivo [23]. Those mice whose tumors had reached a size of 75 to 288 mm^3^ within about 3 weeks of transplantation were orally treated with 40 mg/kg body weight of either CTRL or mebendazole for 5 days per week over a period of 16 days (Figure 4B). Compared to tumors in mice treated with vehicle, mebendazole-treated mice showed a significantly decreased tumor growth (Figure 4C). Of note, the body weight stayed grossly unchanged during the treatment period (Figure 4C), and no changes in physical appearance and behavior were found. Subsequent immunofluorescent staining of the treated tumor specimens displayed a significantly reduced number of Ki67-positive proliferating cells compared to the vehicle treated tumor tissue (Figure 4D). Moreover, histological examination of the treated tumor specimens revealed continuous areas of cell death with apoptotic cells, as evidenced by eosinophilic remnants and condensed nuclei (Figure 4E). Accordingly, these areas stained positive for the apoptotic marker cleaved caspase-3 (Figure 4E). In summary, these experiments clearly show that mebendazole is efficient and safe to be used in chemoresistant and aggressive HB models.

## 4. Discussion

The lack of therapeutic options for HB patients that do not respond well to preoperative chemotherapy is a significant problem. The development of new cancer drugs is hampered by immense costs and the lack of interest of pharmaceutical companies when it comes to rare diseases such as HB. The repositioning of old drugs that already passed safety tests in humans but stalled in clinical trials or others that have been approved for other indications represents an appealing, safe, and cost-effective approach for cancer drug discovery [44]. Advances in technology such as next generation sequencing and computational drug prediction modeling nowadays allow for systematic searches for candidates from these old drugs. CMap represents one such elegant approach. It is a unique platform to connect genes, drugs, and disease by virtue of common gene expression signatures [9,10]. Using this drug prediction tool and RNA sequencing-based gene expression data from HB patients for whom therapy response data were available, we identified mebendazole as a potent inhibitor of tumor growth in both in vitro and in vivo models of chemoresistant HB. Mebendazole is currently recommended by the WHO for treating a range of parasitic worm infections in endemic countries and has already been used in millions of patients with only a few mild side effects. Thus, its repurposing for HB might offer a promising strategy with fewer safety concerns as compared to combinations of conventional chemotherapeutic agents.

Mebendazole is given to adults and children > 2 years to treat roundworm, hookworm, and whipworm infections at a daily dose of 200 mg for 3 consecutive days [45], which corresponds to a daily concentration of approximately 20 mg/kg body weight for a 2-year-old child. Even in populations younger than 24 months, the use of mebendazole has been reported to be safe [46]. In addition, clinical data from long-term mebendazole therapy in the treatment of alveolar echinococcosis suggest that mebendazole exhibits minimal toxicity when used in cyclic and continuous regimens [47]. In our experimental mouse model setting, we used mebendazole in a concentration of 40 mg/kg body weight for 5 days on/2 days off. As evidenced by an unchanged body weight, we observed no adverse side effects to this regimen over a treatment period of 16 days. In line with this, mebendazole has also been safely applied to mice bearing different cancer types including those from lung, brain, skin, kidney, and colon in oral doses of 40–80 mg/kg body weight given 4–7 times per week over a period of 3–4 weeks [48,49,50,51,52]. Of note, mebendazole also reduced tumor initiation in *Apc^Min/+^* mice, a preclinical model for colon cancer with activated Wnt signaling [52]. Collectively, these data indicate a safe side effect profile for mebendazole, even in the pediatric population, and that it can be successfully used in inhibiting in vitro and in vivo tumor growth of a variety of cancers, including HB, at clinically achievable doses.

Encouraged by the positive results of many preclinical studies, first attempts have been made to make mebendazole available for cancer patient treatment in the clinical setting. A first phase 1 trial on newly diagnosed high-grade glioma patients reinforced long-term safety of mebendazole at doses up to 200 mg/kg for 6–12 months with acceptable toxicities [53]. Another trial on therapy refractory gastrointestinal cancer patients with progressive disease had to be prematurely terminated because of the lack of any anti-cancer effect when given as a single drug, thereby indicating that mebendazole should be combined with a cytotoxic drug [54]. Nevertheless, mebendazole was shown to be safe and well-tolerated in this study, even at doses up to 4 g/day, with abdominal pain, decreased appetite, nausea, and vomiting being the most commonly reported adverse side effects. Just recently, mebendazole has proved safety and efficacy in the adjuvant treatment of advanced colon cancer when given in combination with chemotherapy [55]. In a prospective, randomized, placebo-controlled study, Hegazy and colleagues showed that mebendazole dosed in a range between 8.33 and 24.39 mg/kg body weight per day over a time of 12 weeks was well tolerated, with diarrhea and abdominal pain being the only significant adverse side effects compared to the baseline treatment. Computerized tomography scans 12 weeks after intervention revealed that the addition of mebendazole to the standard of care bevacizumab/FOLFOX4 treatment enhanced the overall response rate from 10% to 65% in the control and mebendazole groups, respectively. Although this difference vanished after the median treatment duration of 12 months and thus the difference in the one-year overall survival was not significant, the progression-free survival could be significantly elevated from 3 to 9 months with the addition of mebendazole. Based on its safety and efficacy in enhancing tumor response to chemotherapy, it might be speculated that mebendazole in combination with cisplatin will provide a window of opportunity for currently inoperable chemoresistant HB tumors to be shrunk sufficiently so as to achieve surgical resectability and thus improve prognosis. This is even more likely given the high synergy scores of mebendazole with cisplatin in the cell culture model, which are comparable to the ones achieved by the standard therapy of doxorubicin combined with cisplatin.

The mechanism of action behind the anti-parasitic effect of mebendazole is believed to be mediated by its microtubule-disrupting capability, which prevents the polymerization of tubulin in the gut of helminths, causing them to die [36]. A large body of evidence suggests that mebendazole-mediated microtubule damage in cancer cells results in spindle disruption and thus cell cycle arrest in the G2/M phase, thereby halting cell proliferation and ultimately leading to apoptosis [56]. Congruently, we detected a significant downregulation of tubulins and the aforementioned cellular consequences in our three tested PDX models upon exposure to mebendazole. In addition, our RNA sequencing-based screening approach also identified genes involved in building-up the unwindosome to be heavily affected by mebendazole. This protein complex consists of six MCM subunits arranged in a gapped ring structure, which is closed by CDC45 and GINS, and its assembly on the DNA is responsible for unwinding duplex DNA during DNA replication [40]. Interestingly, *GINS2* has been described to play a major role in the progression of hepatocellular carcinoma, and its knockdown completely phenocopied the cellular consequences on tumor growth, colony formation ability, migration, cell cycle, and apoptosis found by mebendazole treatment in our HB models [57]. In line with this, the silencing of *CDC45* in papillary thyroid cancer cells resulted in identical tumor compromising effects [58]. Most relevantly, the inhibition of MCM complex genes sensitized pancreatic ductal adenocarcinoma and colon carcinoma cells to chemotherapeutic drugs such as 5-fluorouracil, gemcitabine, oxaliplatin, and etoposide [59]. It is worth mentioning that these chemotherapeutic drugs function indirectly or directly by blocking the DNA replication fork, as cisplatin, which is the backbone of HB therapy [7], also does [60]. Our results demonstrating the transcriptional downregulation of unwindosome-constituting genes implied association of their activation in resistance to chemotherapy. Intriguingly, we could corroborate that *MCM2, MCM5, GINS2*, and *CDC45* are highly expressed in tumors that did not respond to chemotherapy. Moreover, unwindosome activation was significantly associated with the adverse C2 subtype of the 16-gene signature [14], the high-risk MRS-3 subtype [15], and poor event-free survival in an independent cohort of HB patients. Taken together, these data clearly establish unwindosome-associated genes as a predictor for therapeutic success and furthermore confer two established prognostic biomarkers a theranostic value. Moreover, this might have important clinical implications as it implies that one could use mebendazole together with cisplatin as combination regimen to substitute for cisplatin and doxorubicin treatments [8] in order to reduce toxic side effects of doxorubicin. In line with our results, a first study has recently demonstrated that mebendazole could overcome cisplatin resistance in ovarian cancer in vitro [61].

## 5. Conclusions

In conclusion, our results suggest that mebendazole could be used in combination with existing treatments as a safe and readily available drug in treating HB patients, especially for those who do not respond to cisplatin alone. Further work needs to be done into optimized dosing and combination schedules, which should be elaborated in a small-scale clinical trial.

## Figures and Tables

**Table 1:** Correlation of unwindosome gene expression with clinical and molecular features.

			*MCM2*			*MCM5*			*GINS2*			*CDC45*	
Characteristic		Down	Up	*p*-Value	Down	Up	*p*-Value	Down	Up	*p*-Value	Down	Up	*p*-Value
Vascular invasion	Yes No	10 5	4 8	0.085	10 5	4 8	0.085	9 5	5 8	0.180	10 5	4 8	0.085
Metastasis	Yes No	10 4	7 5	0.411	11 4	7 5	0.411	10 4	8 5	0.586	11 4	7 5	0.411
Multifocality	Yes No	10 5	5 7	0.194	9 6	9 6	0.603	9 5	6 7	0.343	10 5	5 7	0.194
Age > 8 years	Yes No	14 1	9 3	0.183	14 1	9 3	0.183	13 1	10 3	0.244	14 1	9 3	0.183
PRETEXT ^1^	1, 2, 3 4	11 3	10 2	0.629	11 3	10 2	0.629	10 3	11 2	0.545	11 3	10 2	0.629
Histology	Epithelial Mixed	6 9	8 4	0.168	6 9	8 4	0.168	5 9	9 4	0.082	6 9	8 4	0.168
Main epithelial component	Fetal Non-fetal	10 4	4 8	0.099	10 4	4 8	0.099	10 3	4 9	0.038	10 4	4 8	0.099
CHIC group ^2^	VL, L, I H	10 5	5 7	0.194	10 5	5 7	0.194	9 5	6 7	0.343	10 5	5 7	0.194
16-gene ^3^	C1 C2	10 5	3 9	0.031	11 4	2 10	0.003	10 4	3 10	0.012	10 5	3 9	0.031
MRS ^4^	1 2 3	8 4 3	2 2 8	0.043	8 4 3	2 2 8	0.043	8 4 2	2 2 9	0.013	8 4 3	2 2 8	0.043

^1^ Stage according to pretreatment extent of disease [41]. ^2^ Risk group defined by the Children’s Hepatic tumors International Collaboration [42], with: VL, very low; L, low; I, intermediate; H, high. ^3^ Subgroups according to the 16-gene signature [14], with: C1, cluster 1; C2, cluster 2. ^4^ Molecular risk stratification defined by [15].

## Data Availability

RNA sequencing data for the CMap analysis and for the clinical association study were downloaded from Gene Expression Omnibus with the accession numbers GSE151347 and GSE132219, respectively. RNA sequencing data of mebendazole-treated cell lines produced in this study are available at Gene Expression Omnibus (accession number GSE209824).

## Supplementary Materials

The following supporting information can be downloaded at: https://www.mdpi.com/article/10.3390/cancers14174196/s1, Table S1: Differentially expressed genes of non-responders; Table S2: CMap drug list; Table S3: Differentially expressed genes upon mebendazole treatment; Table S4: Gene ontology enrichment analysis.

## References

[1] Czauderna P., Lopez-Terrada D., Hiyama E., Haberle B., Malogolowkin M.H., Meyers R.L. (2014). Hepatoblastoma state of the art: Pathology, genetics, risk stratification, and chemotherapy. Curr. Opin. Pediatr..

[2] Tomlinson G.E., Kappler R. (2012). Genetics and epigenetics of hepatoblastoma. Pediatr. Blood Cancer.

[3] Eichenmüller M., Trippel F., Kreuder M., Beck A., Schwarzmayr T., Häberle B., Cairo S., Leuschner I., von Schweinitz D., Strom T.M. (2014). The genomic landscape of hepatoblastoma and their progenies with HCC-like features. J. Hepatol..

[4] Hirsch T.Z., Pilet J., Morcrette G., Roehrig A., Monteiro B.J.E., Molina L., Bayard Q., Trepo E., Meunier L., Caruso S. (2021). Integrated Genomic Analysis Identifies Driver Genes and Cisplatin-Resistant Progenitor Phenotype in Pediatric Liver Cancer. Cancer Discov..

[5] Nagae G., Yamamoto S., Fujita M., Fujita T., Nonaka A., Umeda T., Fukuda S., Tatsuno K., Maejima K., Hayashi A. (2021). Genetic and epigenetic basis of hepatoblastoma diversity. Nat. Commun..

[6] Sumazin P., Chen Y., Trevino L.R., Sarabia S.F., Hampton O.A., Patel K., Mistretta T.A., Zorman B., Thompson P., Heczey A. (2017). Genomic analysis of hepatoblastoma identifies distinct molecular and prognostic subgroups. Hepatology.

[7] Perilongo G., Maibach R., Shafford E., Brugieres L., Brock P., Morland B., de Camargo B., Zsiros J., Roebuck D., Zimmermann A. (2009). Cisplatin versus cisplatin plus doxorubicin for standard-risk hepatoblastoma. N. Engl. J. Med..

[8] Zsiros J., Maibach R., Shafford E., Brugieres L., Brock P., Czauderna P., Roebuck D., Childs M., Zimmermann A., Laithier V. (2010). Successful treatment of childhood high-risk hepatoblastoma with dose-intensive multiagent chemotherapy and surgery: Final results of the SIOPEL-3HR study. J. Clin. Oncol..

[9] Lamb J. (2007). The Connectivity Map: A new tool for biomedical research. Nat. Rev. Cancer.

[10] Qu X.A., Rajpal D.K. (2012). Applications of Connectivity Map in drug discovery and development. Drug Discov. Today.

[11] Claerhout S., Lim J.Y., Choi W., Park Y.Y., Kim K., Kim S.B., Lee J.S., Mills G.B., Cho J.Y. (2011). Gene expression signature analysis identifies vorinostat as a candidate therapy for gastric cancer. PLoS ONE.

[12] Hieronymus H., Lamb J., Ross K.N., Peng X.P., Clement C., Rodina A., Nieto M., Du J., Stegmaier K., Raj S.M. (2006). Gene expression signature-based chemical genomic prediction identifies a novel class of HSP90 pathway modulators. Cancer Cell.

[13] Wei G., Twomey D., Lamb J., Schlis K., Agarwal J., Stam R.W., Opferman J.T., Sallan S.E., den Boer M.L., Pieters R. (2006). Gene expression-based chemical genomics identifies rapamycin as a modulator of MCL1 and glucocorticoid resistance. Cancer Cell.

[14] Cairo S., Armengol C., De Reynies A., Wei Y., Thomas E., Renard C.A., Goga A., Balakrishnan A., Semeraro M., Gresh L. (2008). Hepatic stem-like phenotype and interplay of Wnt/beta-catenin and Myc signaling in aggressive childhood liver cancer. Cancer Cell.

[15] Carrillo-Reixach J., Torrens L., Simon-Coma M., Royo L., Domingo-Sabat M., Abril-Fornaguera J., Akers N., Sala M., Ragull S., Arnal M. (2020). Epigenetic footprint enables molecular risk stratification of hepatoblastoma with clinical implications. J. Hepatol..

[16] Hooks K.B., Audoux J., Fazli H., Lesjean S., Ernault T., Dugot-Senant N., Leste-Lasserre T., Hagedorn M., Rousseau B., Danet C. (2018). New insights into diagnosis and therapeutic options for proliferative hepatoblastoma. Hepatology.

[17] Cairo S., Armengol C., Maibach R., Haberle B., Becker K., Carrillo-Reixach J., Guettier C., Vokuhl C., Schmid I., Buendia M.A. (2020). A combined clinical and biological risk classification improves prediction of outcome in hepatoblastoma patients. Eur. J. Cancer.

[18] Wagner A.E., Schwarzmayr T., Haberle B., Vokuhl C., Schmid I., von Schweinitz D., Kappler R. (2020). SP8 Promotes an Aggressive Phenotype in Hepatoblastoma via FGF8 Activation. Cancers.

[19] Haberle B., Maxwell R., Schweinitz D.V., Schmid I. (2019). High Dose Chemotherapy with Autologous Stem Cell Transplantation in Hepatoblastoma does not Improve Outcome. Results of the GPOH Study HB99. Klin. Padiatr..

[20] Love M.I., Huber W., Anders S. (2014). Moderated estimation of fold change and dispersion for RNA-seq data with DESeq2. Genome Biol..

[21] Lamb J., Crawford E.D., Peck D., Modell J.W., Blat I.C., Wrobel M.J., Lerner J., Brunet J.P., Subramanian A., Ross K.N. (2006). The Connectivity Map: Using gene-expression signatures to connect small molecules, genes, and disease. Science.

[22] Pietsch T., Fonatsch C., Albrecht S., Maschek H., Wolf H.K., von Schweinitz D. (1996). Characterization of the continuous cell line HepT1 derived from a human hepatoblastoma. Lab. Investig..

[23] Nicolle D., Fabre M., Simon-Coma M., Gorse A., Kappler R., Nonell L., Mallo M., Haidar H., Deas O., Mussini C. (2016). Patient-derived mouse xenografts from pediatric liver cancer predict tumor recurrence and advise clinical management. Hepatology.

[24] Ianevski A., Giri A.K., Aittokallio T. (2020). SynergyFinder 2.0: Visual analytics of multi-drug combination synergies. Nucleic Acids Res..

[25] Chen W., Wong C., Vosburgh E., Levine A.J., Foran D.J., Xu E.Y. (2014). High-throughput image analysis of tumor spheroids: A user-friendly software application to measure the size of spheroids automatically and accurately. J. Vis. Exp..

[26] Dobin A., Davis C.A., Schlesinger F., Drenkow J., Zaleski C., Jha S., Batut P., Chaisson M., Gingeras T.R. (2013). STAR: Ultrafast universal RNA-seq aligner. Bioinformatics.

[27] Anders S., Pyl P.T., Huber W. (2015). HTSeq—A Python framework to work with high-throughput sequencing data. Bioinformatics.

[28] Ge S.X., Jung D., Yao R. (2020). ShinyGO: A graphical gene-set enrichment tool for animals and plants. Bioinformatics.

[29] Szklarczyk D., Gable A.L., Nastou K.C., Lyon D., Kirsch R., Pyysalo S., Doncheva N.T., Legeay M., Fang T., Bork P. (2021). The STRING database in 2021: Customizable protein-protein networks, and functional characterization of user-uploaded gene/measurement sets. Nucleic Acids Res..

[30] Wagner F., Henningsen B., Lederer C., Eichenmüller M., Gödeke J., Müller-Höcker J., von Schweinitz D., Kappler R. (2012). Rapamycin blocks hepatoblastoma growth in vitro and in vivo implicating new treatment options in high-risk patients. Eur. J. Cancer.

[31] Hartmann W., Kuchler J., Koch A., Friedrichs N., Waha A., Endl E., Czerwitzki J., Metzger D., Steiner S., Wurst P. (2009). Activation of phosphatidylinositol-3′-kinase/AKT signaling is essential in hepatoblastoma survival. Clin. Cancer Res..

[32] Aden D.P., Fogel A., Plotkin S., Damjanov I., Knowles B.B. (1979). Controlled synthesis of HBsAg in a differentiated human liver carcinoma-derived cell line. Nature.

[33] Nakabayashi H., Taketa K., Yamane T., Miyazaki M., Miyano K., Sato J. (1984). Phenotypical stability of a human hepatoma cell line, HuH-7, in long-term culture with chemically defined medium. Gan.

[34] Eloranta K., Cairo S., Liljestrom E., Soini T., Kyronlahti A., Judde J.G., Wilson D.B., Heikinheimo M., Pihlajoki M. (2020). Chloroquine Triggers Cell Death and Inhibits PARPs in Cell Models of Aggressive Hepatoblastoma. Front. Oncol..

[35] Zsiros J., Brugieres L., Brock P., Roebuck D., Maibach R., Zimmermann A., Childs M., Pariente D., Laithier V., Otte J.B. (2013). Dose-dense cisplatin-based chemotherapy and surgery for children with high-risk hepatoblastoma (SIOPEL-4): A prospective, single-arm, feasibility study. Lancet Oncol..

[36] Laclette J.P., Guerra G., Zetina C. (1980). Inhibition of tubulin polymerization by mebendazole. Biochem. Biophys. Res. Commun..

[37] Draber P., Draberova E. (2021). Dysregulation of Microtubule Nucleating Proteins in Cancer Cells. Cancers.

[38] Seibert M., Kruger M., Watson N.A., Sen O., Daum J.R., Slotman J.A., Braun T., Houtsmuller A.B., Gorbsky G.J., Jacob R. (2019). CDK1-mediated phosphorylation at H2B serine 6 is required for mitotic chromosome segregation. J. Cell Biol..

[39] Musacchio A., Salmon E.D. (2007). The spindle-assembly checkpoint in space and time. Nat. Rev. Mol. Cell Biol..

[40] Seo Y.S., Kang Y.H. (2018). The Human Replicative Helicase, the CMG Complex, as a Target for Anti-cancer Therapy. Front. Mol. Biosci..

[41] Roebuck D.J., Aronson D., Clapuyt P., Czauderna P., de Ville de Goyet J., Gauthier F., Mackinlay G., Maibach R., McHugh K., Olsen O.E. (2007). 2005 PRETEXT: A revised staging system for primary malignant liver tumours of childhood developed by the SIOPEL group. Pediatr. Radiol..

[42] Meyers R.L., Maibach R., Hiyama E., Haberle B., Krailo M., Rangaswami A., Aronson D.C., Malogolowkin M.H., Perilongo G., von Schweinitz D. (2017). Risk-stratified staging in paediatric hepatoblastoma: A unified analysis from the Children’s Hepatic tumors International Collaboration. Lancet Oncol..

[43] Jamieson L.E., Harrison D.J., Campbell C.J. (2015). Chemical analysis of multicellular tumour spheroids. Analyst.

[44] Nosengo N. (2016). Can you teach old drugs new tricks?. Nature.

[45] Bethony J., Brooker S., Albonico M., Geiger S.M., Loukas A., Diemert D., Hotez P.J. (2006). Soil-transmitted helminth infections: Ascariasis, trichuriasis, and hookworm. Lancet.

[46] Montresor A., Awasthi S., Crompton D.W. (2003). Use of benzimidazoles in children younger than 24 months for the treatment of soil-transmitted helminthiasis. Acta Trop..

[47] Reuter S., Jensen B., Buttenschoen K., Kratzer W., Kern P. (2000). Benzimidazoles in the treatment of alveolar echinococcosis: A comparative study and review of the literature. J. Antimicrob. Chemother..

[48] Bai R.Y., Staedtke V., Aprhys C.M., Gallia G.L., Riggins G.J. (2011). Antiparasitic mebendazole shows survival benefit in 2 preclinical models of glioblastoma multiforme. Neuro Oncol..

[49] Doudican N.A., Byron S.A., Pollock P.M., Orlow S.J. (2013). XIAP downregulation accompanies mebendazole growth inhibition in melanoma xenografts. Anticancer Drugs.

[50] Martarelli D., Pompei P., Baldi C., Mazzoni G. (2008). Mebendazole inhibits growth of human adrenocortical carcinoma cell lines implanted in nude mice. Cancer Chemother. Pharmacol..

[51] Mukhopadhyay T., Sasaki J., Ramesh R., Roth J.A. (2002). Mebendazole elicits a potent antitumor effect on human cancer cell lines both in vitro and in vivo. Clin. Cancer Res..

[52] Williamson T., Bai R.Y., Staedtke V., Huso D., Riggins G.J. (2016). Mebendazole and a non-steroidal anti-inflammatory combine to reduce tumor initiation in a colon cancer preclinical model. Oncotarget.

[53] Gallia G.L., Holdhoff M., Brem H., Joshi A.D., Hann C.L., Bai R.Y., Staedtke V., Blakeley J.O., Sengupta S., Jarrell T.C. (2021). Mebendazole and temozolomide in patients with newly diagnosed high-grade gliomas: Results of a phase 1 clinical trial. Neurooncol. Adv..

[54] Mansoori S., Fryknas M., Alvfors C., Loskog A., Larsson R., Nygren P. (2021). A phase 2a clinical study on the safety and efficacy of individualized dosed mebendazole in patients with advanced gastrointestinal cancer. Sci. Rep..

[55] Hegazy S.K., El-Azab G.A., Zakaria F., Mostafa M.F., El-Ghoneimy R.A. (2022). Mebendazole; from an anti-parasitic drug to a promising candidate for drug repurposing in colorectal cancer. Life Sci..

[56] Guerini A.E., Triggiani L., Maddalo M., Bonu M.L., Frassine F., Baiguini A., Alghisi A., Tomasini D., Borghetti P., Pasinetti N. (2019). Mebendazole as a Candidate for Drug Repurposing in Oncology: An Extensive Review of Current Literature. Cancers.

[57] Zhang Y., Hao X., Han G., Lu Y., Chen Z., Zhang L., Wu J., Wang X. (2022). E2F1-mediated GINS2 transcriptional activation promotes tumor progression through PI3K/AKT/mTOR pathway in hepatocellular carcinoma. Am. J. Cancer Res..

[58] Sun J., Shi R., Zhao S., Li X., Lu S., Bu H., Ma X. (2017). Cell division cycle 45 promotes papillary thyroid cancer progression via regulating cell cycle. Tumour. Biol..

[59] Bryant V.L., Elias R.M., McCarthy S.M., Yeatman T.J., Alexandrow M.G. (2015). Suppression of Reserve MCM Complexes Chemosensitizes to Gemcitabine and 5-Fluorouracil. Mol. Cancer Res..

[60] Kelland L. (2007). The resurgence of platinum-based cancer chemotherapy. Nat. Rev. Cancer.

[61] Huang L., Zhao L., Zhang J., He F., Wang H., Liu Q., Shi D., Ni N., Wagstaff W., Chen C. (2021). Antiparasitic mebendazole (MBZ) effectively overcomes cisplatin resistance in human ovarian cancer cells by inhibiting multiple cancer-associated signaling pathways. Aging.

